# Gut, Microbiome, and Brain Regulatory Axis: Relevance to Neurodegenerative and Psychiatric Disorders

**DOI:** 10.1007/s10571-018-0589-2

**Published:** 2018-05-25

**Authors:** G. B. Stefano, N. Pilonis, R. Ptacek, J. Raboch, M. Vnukova, R. M. Kream

**Affiliations:** 1 Department of Psychiatry, First Faculty of Medicine Charles University in Prague and General University Hospital in Prague, Center for Cognitive and Molecular Neuroscience, Ke Karlovu 11, 120 00 Prague 2, Czech Republic; 2 Warsaw Medical University, Public Central Teaching Hospital, Warsaw, Poland

**Keywords:** Microbiome, Psychiatry, Depression, Bacteria, Antibiotics, Monoamines, FOXG1

## Abstract

It has become apparent that the molecular and biochemical integrity of interactive families, genera, and species of human gut microflora is critically linked to maintaining complex metabolic and behavioral processes mediated by peripheral organ systems and central nervous system neuronal groupings. Relatively recent studies have established intrinsic ratios of enterotypes contained within the human microbiome across demographic subpopulations and have empirically linked significant alterations in the expression of bacterial enterotypes with the initiation and persistence of several major metabolic and psychiatric disorders. Accordingly, the goal of our review is to highlight potential thematic/functional linkages of pathophysiological alterations in gut microbiota and bidirectional gut–brain signaling pathways with special emphasis on the potential roles of gut dysbiosis on the pathophysiology of psychiatric illnesses. We provide critical discussion of putative thematic linkages of Parkinson’s disease (PD) data sets to similar pathophysiological events as potential causative factors in the development and persistence of diverse psychiatric illnesses. Finally, we include a concise review of preclinical paradigms that involve immunologically–induced GI deficits and dysbiosis of maternal microflora that are functionally linked to impaired neurodevelopmental processes leading to affective behavioral syndromes in the offspring.

## Introduction

A key goal of biomedical research is to identify a common set of causative factors that is directly linked to pathophysiological changes observed in major neurodegenerative diseases afflicting human populations. A hallmark example identifies the major etiological factor in Parkinson’s disease (PD) as a relatively slow temporal loss of striatal dopaminergic (DA-ergic) transmission, resulting from a degeneration of neuronal somata located in the substantia nigra (Fearnley and Lees 1991). Furthermore, a wealth of published biochemical, cellular, and molecular studies, is focused on pathophysiological changes in mitochondrial function linked to diminished cellular bioenergetics, free radical generation, impaired protein transport and metabolism, and diminished mitochondrial biogenesis as key causative factors in PD development (Giannoccaro et al. 2017; McWilliams and Muqit 2017; Smith et al. 2017; Winklhofer and Haass 2010). Currently, a burst of empirical investigation and critical thinking has focused on elucidating functional aspects of the intestinal microbiota in maintaining ongoing physiological processes within the gastrointestinal (GI) tract (Hyland and Cryan 2016; Obata and Pachnis 2016; Yano et al. 2015). Of equivalent importance, the integrity of bidirectional gut–brain signaling pathways appears to be dependent on a functionally active, healthy, microbiome (Diaz Heijtz et al. 2011; Zhou and Foster 2015) and dysbiosis of the human gut microbiota has been functionally associated with central nervous system (CNS) degenerative disorders that include PD (Bedarf et al. 2017; Devos et al. 2013; Forsyth et al. 2011; Malkki 2017; Mulak and Bonaz 2015; Rietdijk et al. 2017; Scheperjans et al. 2015), Alzheimer disease, Huntington disease, and amyotrophic lateral sclerosis (Main and Minter 2017; Tremlett et al. 2017).

The goal of this review is to highlight potential thematic/functional linkages of pathophysiological alterations in gut microbiota and bidirectional gut–brain signaling pathways with special emphasis on the potential roles of gut dysbiosis on the pathophysiology of psychiatric illnesses. We provide a concise discussion of evolutionarily conserved, intrinsic DA-ergic, 5-HT-ergic, and GABA-ergic signaling pathways between gut microbiota, enteric neurons, and enterochromaffin (EC) cells, that partially mediate homeostasis of innate immunity (Petra et al. 2015; Reigstad et al. 2015; Scheperjans et al. 2015; Stefano et al. 1976). This is followed by a concise review of comorbid pathophysiological events preceding observed motor symptoms associated with PD that include concerted dysregulation of GI function via dysbiosis of gut microbiota, loss of intrinsic ENS/lymphoid coupling, severely diminished immune competence, and progressive colonic inflammation. Furthermore, we provide critical discussion of putative thematic linkages of PD data sets to similar pathophysiological events as potential causative factors in the development and persistence of diverse psychiatric illnesses. Finally, we include a concise review of preclinical paradigms that involve immunologically induced GI deficits and dysbiosis of maternal microflora that are functionally linked to impaired neurodevelopmental processes leading to affective behavioral syndromes in the offspring.

## Biogenic Amine Signaling Linkages of Gut Microbiota, Neurons, and Lymphoid Tissues

We have previously contended that the retention of a core of archetypal chemical messengers across animal and plant phyla represents a likely mechanistic driving force for evolutionary expansion of complex cellular systems (Stefano 1986; Stefano and Kream 2007, 2010). The biological elegance of dopamine (DA; 3,4-dihydroxyphenylethylamine) resides in its pivotal role as a prototype chemical mediator of diverse signaling and metabolic events including locomotor behaviors across a wide spectrum of animal phyla (Kutchko and Siltberg-Liberles 2013; Rivard et al. 2010; Stefano et al. 1976; Stefano and Kream 2007). Although studies designed to directly assess the regulatory roles of free DA released within the lumen of the gut remain scarce (Roshchina 2010), it appears that the contributions of several species of microbiota to maintain normative DA concentrations are of critical importance to maintenance of innate immunity involving the enteric nervous system (ENS) and mucosal lymphoid tissues (Hyland and Cryan 2016; Mittal et al. 2017; Mulak and Bonaz 2015; Obata and Pachnis 2016; Yoo and Mazmanian 2017). Interestingly, a recent preclinical model of antibiotic-induced dysbiosis linked to severe ENS damage and impaired GI function in young mice raises significant concern relating to widespread usage of antibiotics in children and potential dysregulation of bidirectional gut–CNS signaling systems in later life (Caputi et al. 2017; Stefano et al. 2017).

Serotonin (5-hydroxytryptamine or 5-HT) is a multifunctional biogenic amine with neuronal and endocrine signaling roles in a range of GI physiological pathways (Martin et al. 2017). Over 90% of whole body 5-HT is synthesized within populations of EC cells and neurons of the ENS distributed throughout superficial and deeper layers of the GI tract. EC cells appear to integrate nutritional cues with ENS activity and gut microbiome homeostasis via signaling processes mediated by multiple 5-HT receptor types and subtypes (Martin et al. 2017; Mawe and Hoffman 2013). Dysregulation of integrated 5-HT signaling pathways within the gut has been functionally linked to an array of pathophysiological conditions (Mawe and Hoffman 2013).

Within the gut, biosynthesis of 5-HT within EC cells and ENS neurons is catalyzed by 2 isozymes of tryptophan hydroxylase (Tph), Tph1 and Tph2, respectively (Martin et al. 2017). Furthermore, biosynthesis of chemically authentic 5-HT has been demonstrated in the following strains of gut microflora: *Lactococcus lactis, Lactobacillus plantarum, Streptococcus thermophiles, Escherichia coli* K-12, *Morganella morganii, Klebsiella pneumonia, Hafnia alvei* (Özoğul 2004; Shishov et al. 2009). The bacterial production of 5-HT occurs independently of tryptophan hydroxylase (THPH) via decarboxylation of tryptophan to tryptamine, as seen in plants (Shishov et al. 2009). Temporally and spatially defined release of 5-HT from EC cells, ENS neurons, and gut microbiota may differentially activate 14 different 5-HT receptor subtypes located on ENS neurons and immune cells. Furthermore, blood borne 5-HT evolving from the GI tract is sequestered by circulating platelets, maintaining hemostasis via platelet aggregation that ultimately affects immune competence, bone marrow development and cardiac function (Baganz and Blakely 2013; Gershon and Tack 2007; Hoffman et al. 2012; Mawe and Hoffman 2013). Importantly, pathogenic strains of *E. coli* have been observed to inhibit intestinal 5-HT transporter function and expression, thereby confirming the critical role of reciprocal 5-HT signaling processes within the gut (Esmaili et al. 2009).

It has been previously demonstrated that gut microflora partially regulate 5-HT biosynthesis by host cells (Yano et al. 2015). Notably, short chain fatty acids (SCFA) evolving from fermentation processes of gut enterotypes such as Prevotella copri may induce Tph1 gene expression by EC cells (Reigstad et al. 2015). Additionally, commensal bacterial enterotypes such as Bifidobacterium infantis, can modulate tryptophan metabolism and influence the essential precursor pool for 5-HT production within the GI system (Diaz Heijtz et al. 2011). Regulation of 5-HT transporter (SERT) gene expression in the intestinal epithelium has been shown to differ from that of neuronal SERT gene expression (Hoffman et al. 2012; Mawe and Hoffman 2013) and infection with enteropathogenic strains of *E coli* alters SERT gene expression in vivo. These observations provide a potential model for investigating the regulation of SERT as an effective pharmacological target for several GI disorders (Esmaili et al. 2009; Li and Young 2013) and comorbid CNS behavioral syndromes that are partially dependent on altered gut–brain signaling processes.

Gamma-aminobutyric acid (GABA) expression within the gut mediates critical regulatory activities between the ENS and lymphoid tissues involved in immune competence via T cell responses (De Biase and Pennacchietti 2012). Contrary to its marked role within CNS structures, GABA mediates neuronal excitability within the ENS via activation of GABA-GABA_A_ receptor systems (De Biase and Pennacchietti 2012; Hyland and Cryan 2016). Infective microbiota, including *E. coli, Listeria monocytogenes, Shigella flexneri*, and *Lactococcus lactis* depend for their transit through the digestive system on the glutamate-dependent acid resistance mechanism (GDAR). Intraluminal GABA production and release by commensal lactobacilli provides an essential protective mechanism against infective bacteria via GDAR inhibition. In effect, selective strains of commensal microbiota may differentially influence ENS activity via diverse GABA-ergic mechanisms (Hyland and Cryan 2016).

## Pro-inflammatory Processes Initiated by Alterations in Gut Signaling Pathways

**Fig. 1:**
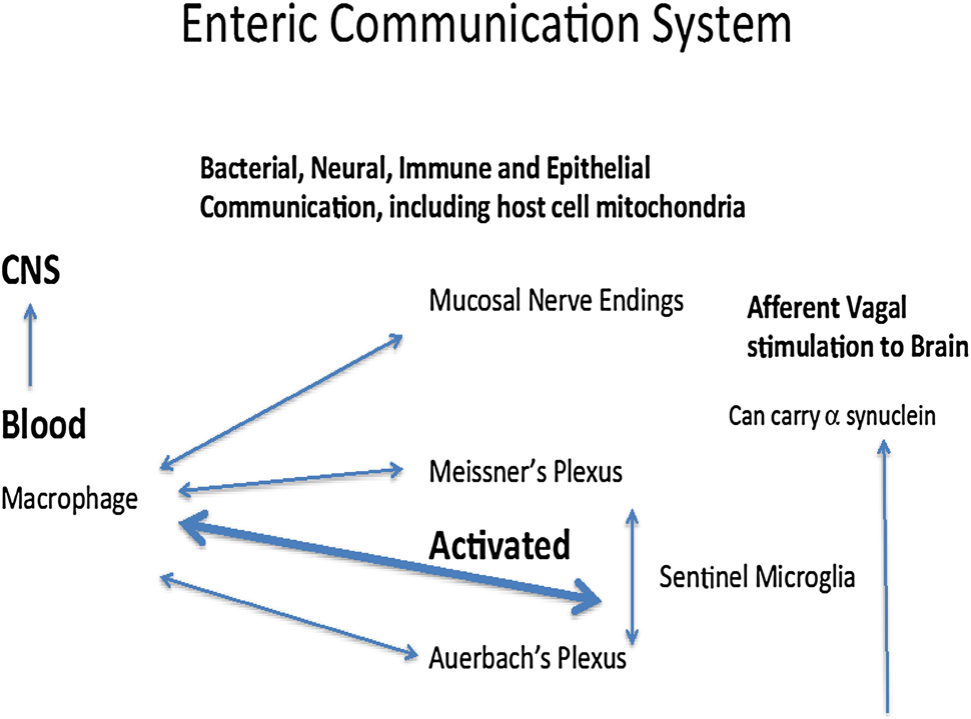
Bacterial intrinsic ratio perturbations are normal occurrences in the microbiome. In part, this phenomenon is monitored by the enteric microglia found in Auerbach’s and Meissner’s plexus. Macrophages freely travel via the circulatory system and gain entrance into the enteric nervous system and reside there fulfilling their sentinel function by transforming into microglia as also occurs in the brain. Clearly, as in the brain, various chemical messengers (e.g., LPS from bacteria) or toxins can activate these cellular sentinels turning them back into macrophages to meet the micro-environmental challenge. Activated macrophages originating from the enteric neuroimmune system may also enter the brain and by so doing activate central nervous system neurons and microglia. For example, the nucleus tractus solitarius is especially susceptible. This abnormal or normal activation may exert profound influences on regions of the brain, controlling our mental processes and consciousness because these activities are always on and thus subject to immediate modification. It is equally important to note that eukaryotic cell mitochondria may be part of this intricate communication between the host and the micro biota. Given the origin of mitochondria as a prokaryotic cell, its ability to communicate bidirectionally with a bacterium also exists and occurs

In the preceding section, we have highlighted the biological necessity of maintaining reciprocal communication processes within microenvironments of the gut via retention and selective adaptations of biogenic amine signaling pathways. As depicted in Fig. 1, commensal enterotypes situated at the luminal surface of the gut mediate essential regulatory activities that coordinate neural and immune function by populations of enterochromaffin cells and ENS neurons via usage of common signal molecules and cells (de Muinck et al. 2017; Stefano et al. 1994). Physiological perturbations of normal gut functioning require minute readjustments of this defined regulatory loop to achieve basal levels of signaling activity with appropriate restoration of intrinsic ratios of microbiota.

Conversely, acute and chronic pathophysiological disruptions of this short intraluminal regulatory loop are functionally linked to severe dysfunction of multiple cell types and microflora, ultimately compromising ENS neuronal activities within the submuscosal and muscularis layers of the colon. Accordingly, a focal point of homeostatic maintenance of essential activities of interactive cell types and microflora rests on the integrity of tight junctions (TJs) situated at the apical surface of adjacent epithelial cells within the lumen of the gut (Coleman and Haller 2017; Wells et al. 2017). Pathophysiological alterations of barrier permeability have been demonstrated to initiate cascades of pro-inflammatory insults to intrinsic cells within superficial and deeper layers, with severe consequences to normal bidirectional gut-brain signaling processes. The essential roles of specific TJ proteins on maintaining normative epithelial permeability, notably zona occludens proteins 1–3, have been demonstrated (Ivanov 2012) and TJ dysfunction has been associated with hyper-permeability functionally linked to the induction and overexpression of key inflammatory cytokines including interferon-gamma, TNF-alpha, IL-1beta, and IL-17 (Manoharan et al. 2016) and underepressiion of anti-inflammatory IL-10 (Ray and Dittel 2015). Furthermore, loss of epithelial barrier integrity is critically linked to perturbation of immune homeostasis and normative generation of regulatory T (Treg) cells from activated CD103^+^ dendritic cells (DCs), resulting in enhanced inflammatory Th1/Th17 responses that are reciprocally linked to severe dysbiosis of commensal microbiota (Barthels et al. 2017; Omenetti and Pizarro 2015).

Production of immune modulators due to pro-inflammatory events is compounded by metabolic insufficiencies linked to Type II diabetes and/or inadequate dietary regimens (Noble et al. 2017) or diminished SCFA production and release by dysbiotic microflora (Kelly et al. 2015; Tulstrup et al. 2015). Resultant downregulation and production of luminal secretory immunoglobulin A (sIgA) and antimicrobial peptides and proteins (AMPs) permit passage of pathogenic microorganisms and pro-inflammatory toxins such as lipopolysaccharide (LPS) through a compromised epithelial barrier into the submucosa and peripheral circulation (Coleman and Haller 2017; Wells et al. 2017). Finally, a recent preclinical study utilizing zebrafish as a developmental model system, has demonstrated that genetic modulation of ENS function due to a mutation in the Hirschsprung disease gene, sox10, results in microbiota-dependent inflammation (Rolig et al. 2017). Enhanced pro-inflammatory processes were associated with altered intrinsic ratios of pro-inflammatory to anti-inflammatory bacterial lineages, thereby demonstrating a critical role of normative ENS physiology to maintain a healthy gut microbiome.

**Fig. 2:**
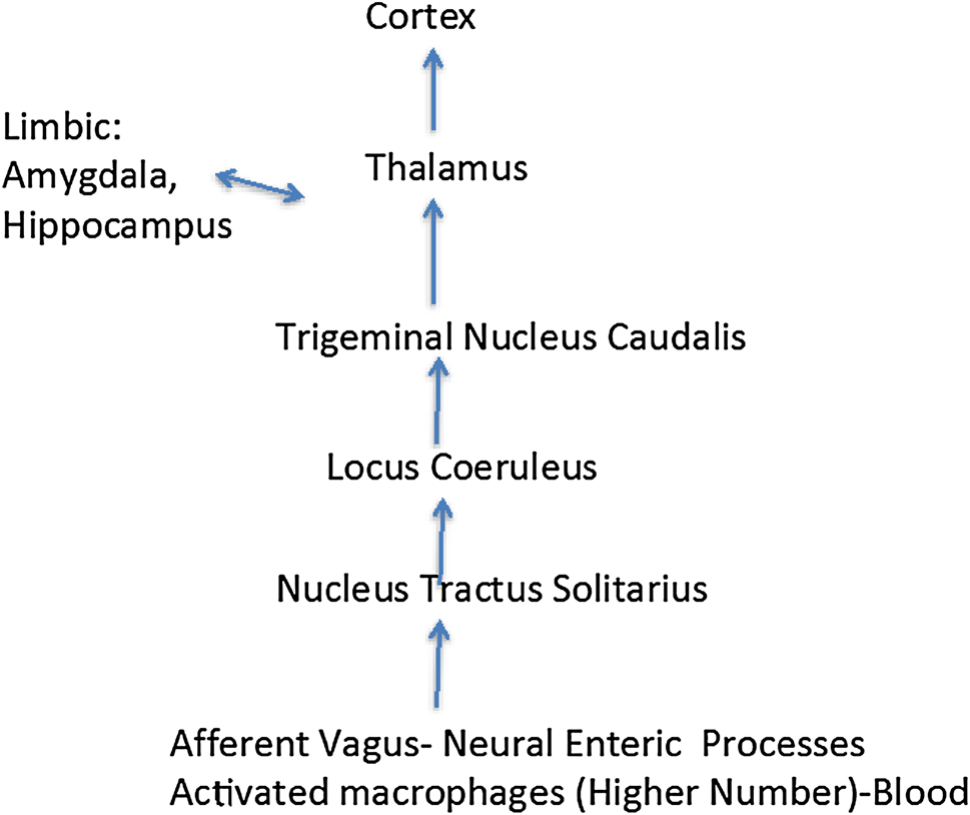
By way of vagal enteric stimulation normal neural pathways may also be susceptible to abnormal microglial conversions into macrophages, inducing enhanced excitation at the peripheral level (e.g., enteric plexi). In this instance, this rapid neural response may also manifest itself via the nucleus tractus solitarius and locus coeruleus, which would influence the amygdala, hippocampus and the thalamus, and ultimately the cortex

It is apparent that the host organism has evolved mechanisms to sense when potentially deleterious perturbations of gut physiology may lead to chronic pathophysiological states (Verheijden et al. 2015) (Fig. 1). In part, these monitoring processes may occur by activation of enteric microglia found in Auerbach’s and Meissner’s plexuses that are key players in the maintenance of innate immunity (Verheijden et al. 2015). Local sustained release of pro-inflammatory mediators within microenvironments of the gut provides a switching mechanism for converting enteric microglia into active immune cells with macrophage-like phenotypic expression (Sonetti et al. 1994; Stefano et al. 1994). Interestingly, circulating macrophages may gain entrance into the ENS and reside there fulfilling their innate immune function via transformation into enteric microglia (Verheijden et al. 2015). Similar to ongoing CNS immune processes, various chemical messengers (e.g., lipopolysaccharide released from circulating bacteria or gut microbiota) can activate these cellular sentinels turning them back into macrophages in order to adequately address micro-environmental challenges (Le et al. 2001; Stefano et al. 1994; Verheijden et al. 2015). We contend that prolonged pro-inflammatory stimuli activate greater numbers of activated microglia to yield enhanced concentrations of circulating macrophages. As previously noted (Stefano et al. 1994), enhanced populations of circulating macrophages that have originated from the enteric immune and nervous systems may ultimately activate CNS neurons and glia. For example, the nucleus tractus solitarius (NTS) of the brainstem is especially susceptible to immune influences with resultant aberrant modulation of essential autonomic regulatory activities (Dworak et al. 2014) (Fig. 2). Rostral transmission of aberrant NTS activation may exert profound influences on regions of the brain regulating integrated cognitive and behavioral processes (Bieri et al. 2017; Lamberts et al. 2015). Additionally, alterations of normative neural-immune signaling processes within the gut–brain axis may be susceptible to poor dietary regimens via modulation of essential ratios of gut microbiota.

## Dysregulation of Gut Signaling Processes as Potential Etiological Factors in Parkinson’s Disease

Comorbid pathophysiological events preceding observed motor symptoms associated with PD include concerted dysregulation of GI function via dysbiosis of gut microbiota, loss of intrinsic ENS/lymphoid coupling, severely diminished immune competence, and progressive colonic inflammation (Bodea et al. 2014; Braak et al. 2003; Dobbs et al. 1999; Forsyth et al. 2011; Kosikowska et al. 2016; Lebouvier et al. 2009; Rietdijk et al. 2017; Villaran et al. 2010). In light of the above, chronic expression of activated macrophages within the submucosal and muscularis layers of the colon may mediate profound degenerative changes within neurons and glia of the ENS (Fig. 1). Accordingly, it has been hypothesized that the slow progression of degenerative changes in the gut is coordinately transmitted via long peripheral nerve and humoral regulatory loops into the CNS, with resultant degeneration of DA-ergic somata of the nigrostriatal CNS pathway (Braak and Del Tredici 2017; Braak et al. 2003; Rietdijk et al. 2017). Recent studies have indicated that PD progression involves pathophysiological disruption of sensory input into the NTS via unmyelinated vagal fibers (Bieri et al. 2017; Lamberts et al. 2015) and subsequent noradrenergic transmission emanating from the locus coeruleus (LC) in the brainstem (Braak and Del Tredici 2017; Vermeiren and De Deyn 2017) (Fig. 2). Furthermore, in a preclinical rat model of PD, vagal nerve stimulation was observed to significantly enhance locomotor activity that was accompanied by increased tyrosine hydroxylase expression and decreased neuroinflammation in the nigrostriatal system (Farrand et al. 2017). Finally, closer examination of the putative gut–CNS anatomical pathway outlined in the original hypothesis of PD induction by Braak and coworkers (Braak et al. 2003) may provide us with a working model of a normative bidirectional anterograde and retrograde signaling pathway linking limbic and cortical CNS groupings with gut microbiota and the ENS.

Several studies have attempted to functionally link progressive stages of PD with changes in intrinsic ratios of gut microbiota (Scheperjans et al. 2015). Notable associations include marked reductions in the abundance of Prevotellaceae/Prevotella species and Bacteroidaceae/Bacteroides species in fecal specimens of PD patients (Scheperjans et al. 2015) and patients displaying active symptoms of inflammatory bowel syndrome [IBS, (Mertsalmi et al. 2017)], as compared to healthy controls. Conversely, increases in the relative abundance of Enterobacteriaceae members were positively correlated with the severity of PD-related motor deficits (Bedarf et al. 2017; Scheperjans et al. 2015), comorbid inflammatory processes (Mertsalmi et al. 2017; Unger et al. 2016), and observable decreases in SCFA production by affected microbiota (Unger et al. 2016).

Interestingly, concerted studies designed to associate established dietary patterns with alterations in Bacteroides and Prevotella enterotypes may provide a functional handle linking the observed changes in species of gut microbiota of PD patients to chronic pathophysiological events involved in disease progression (Wu et al. 2013, 2011). Differential expression of Bacteroides versus Prevotella enterotypes was strongly associated with proto-Western diets enriched in protein and animal fat as compared to proto-agrarian diets enriched in simple and conjugated carbohydrates, respectively (Wu et al. 2011). Accordingly, the putative pathophysiological role of environmentally determined deficiencies of dietary components on altering essential ratios of human gut microbiota, notably Prevotella and Bacteroides oligotypes, appears to be intimately associated with pro-inflammatory processes underlying IBS and PD progression (Wu et al. 2013).

## Comorbid Etiological Factors in Impaired Gut Physiology of Patients Afflicted with Affective Disorders

As discussed above, lessons learned from concerted PD studies of comorbid alterations in gut physiology, dysbiosis of commensal microbiota, and degenerative changes in ENS neurons and glia may be judiciously applied to investigation of diverse psychiatric illnesses. Critical assessment of PD data sets may provide us with putative thematic linkages to evaluate similar pathophysiological events as potential causative factors in the development of Affective Spectrum Disorders (AfSD). Prefrontal cortical and hippocampal regions are densely innervated by rostral noradrenergic projections from LC neurons and have been established to mediate complex cognitive behaviors that are effectively compromised in neurodegenerative and psychiatric diseases (Borodovitsyna et al. 2017). Recent clinical studies confirm the efficacy of vagal nerve stimulation for major depressive disorder (Carreno and Frazer 2017; Lucas et al. 2017; Salloum et al. 2017) and AfSD (Jin and Kong 2017) as mediated by sensory activation of the NTS of the brainstem that directly projects to LC neurons (Beaumont et al. 2017; Yakunina et al. 2017). We therefore contend that a convergence of multidisciplinary studies suggests the existence of bidirectional gut–brain signaling pathways dependent upon efferent vagal/ENS transmission linked to reciprocal sensory vagal stimulation of discrete brainstem nuclei that project to rostral CNS areas involved with cognition and complex behaviors (Fig. 2). Finally, we postulate that dysregulation of bidirectional gut–brain signaling is critically linked to the etiology of AfSD and other psychiatric illnesses.

Several lines of preclinical and clinical evidence have made strong case for the involvement of dysregulated GI function with associated colonic inflammation and dysbiosis of gut microbiota in the etiology and progression of AfSD and related psychiatric disorders (Diaz Heijtz et al. 2011; Frohlich et al. 2016; Petra et al. 2015; Zheng et al. 2016; Zhou and Foster 2015). Furthermore, collected studies from the laboratories of Cryan, Dinan, and collaborators, have provided a mechanistic framework for evaluating the effects of dysbiotic microflora and gut inflammation on altered vagal signaling leading to induction and persistence of CNS behavioral disorders (Borre et al. 2014; Dinan and Cryan 2017; Dinan et al. 2015; Kelly et al. 2016; Sherwin et al. 2016). As discussed earlier, it appears that altered vagal outflow is functionally linked to cascading pro-inflammatory processes driven by pathophysiological alterations in the luminal epithelial barrier (Coleman and Haller 2017; Wells et al. 2017). Furthermore, dysbiosis of gut microbiota mediates significant alterations in 5-HT signaling and tryptophan metabolism (O’Mahony et al. 2015), leading to hyper-excitability of vagal fibers, dysregulation of enterochromaffin cells, and ENS activity (Hyland and Cryan 2016). Finally, a recent preclinical study from this group sought to evaluate the effects of altered vagal activity on brain-derived neurotrophic factor (BDNF) expression in the hippocampus (O’Leary et al. 2018). The authors observed that vagotomy decreased BDNF mRNA expression throughout mouse hippocampus and was associated impaired development of immature neurons displaying complex dendritic morphology. The authors concluded that vagal nerve activity influences neurogenesis via BDNF expression in the hippocampus, thereby providing a cogent model for pathophysiological downregulation of neural plasticity that is associated with severe alterations in the gut-brain-microbiome bidirectional signaling pathway.

In light of these findings, analysis of fecal samples from children with autistic symptoms revealed augmented levels of three Clostridium clusters and one Clostridium species, *C. bolteae*, consistent with chronic dysbiosis of the microbiome (Shaw 2016; Song et al. 2004). High concentrations of Clostridia metabolites and bacterially expressed chemical toxins such as p-cresol have also been associated with various psychiatric disorders and PD (Persico and Napolioni 2013; Shaw 2016). In this instance, Clostridia metabolites have the ability to inhibit dopamine-beta-hydroxylase, increasing DA levels, and oxidative molecules damaging neuronal mitochondria (Shaw 2016). Furthermore, ingestion of herbicide-derived glyphosate has observed to promote toxic dysbiosis of gut microbiota associated with adverse psychiatric symptoms (Shaw 2016; Song et al. 2004). From a biochemical perspective, bacterial homologs of eukaryotic plasma membrane-associated monoamine transporters appear to play a functional role in the disposition and metabolism of DA and 5-HT within the lumen and mucosal layers of the gut (Yamashita et al. 2005). Dysbiosis of gut microbiota associated with altered regulation of monoamine metabolism has been implicated in neurological and neuropsychiatric illnesses (Singh and Pal 2015). Relatively recent clinical and preclinical studies demonstrate limited efficacies of probiotic treatments (Kang et al. 2017; Liu et al. 2016; Wallace and Milev 2017b) and fecal microbiota transplants (Kang et al. 2017) on reduction of adverse behavioral symptoms associated with AfSD.

From a neurodevelopmental perspective, a recent preclinical rodent study has demonstrated that trans-placental exposure of the fetal brain to circulating bacterial peptidoglycan released from the maternal gut microbiome mediates cognitive and behavioral disorders in the offspring (Humann et al. 2016). The authors hypothesize that that moderate to severe dysregulation of maternally derived innate immunity that is functionally associated with dysbiosis of gut microbiota results in release of a potent pro-inflammatory mediator, i.e., bacterial peptidoglycan, that profoundly affects cortical development of the fetal brain via disruption of FOXG1 gene expression (Bodea et al. 2014; Humann et al. 2016). The FOXG1 gene encodes a brain-specific transcriptional activator protein, which plays an important role in cortical CNS development (De Filippis et al. 2012) and animal offspring, so exposed, exhibit decreased cognitive function. Furthermore, it has been empirically demonstrated that FOXG1 overexpression may initiate a dysregulation of GABA/glutamate neuronal differentiation and overproduction of GABA-ergic inhibitory neurons, thereby contributing to established symptomatology associated with AfSD (Mariani et al. 2015). Employment of a different preclinical mouse model has linked immunologically induced maternal GI barrier defects with resulting neuro-developmentally linked behavioral disorders in the offspring (Hsiao 2013). The study demonstrates that oral treatment of behaviorally impaired offspring with strains of human commensal Bacteroides fragilis altered ratios of gut microbiota with amelioration of defects in communicative and stereotypic sensorimotor behaviors.

Finally, dysbiosis of essential strains of gut microflora engendered by dysregulation of biogenic amine signaling pathways may negatively affect essential gut–CNS communication processes at multiple physiological check points that modulate mitochondrial bioenergetics (Petra et al. 2015; Reigstad et al. 2015; Scheperjans et al. 2015; Snyder et al. 2015; Wallace and Milev 2017a; Zheng et al. 2016; Zhou and Foster 2015). Recent studies have attempted to elucidate functional associations of impaired mitochondrial function (Stefano and Kream 2016; Tobe 2013) and/or dysbiosis of gut microflora in the etiology and/or progression of diverse psychiatric and related medical conditions subsumed under the general rubric of AfSD (Thompson et al. 2015; Tobe 2013). Accordingly, the predominant focus of the multi-generation pharmacopeia of clinically employed therapeutic agents for AfSD-related psychiatric disorders involves functional targeting of dysregulated DA-ergic and interactive biogenic amine signaling systems in cortical and limbic CNS areas (Moonen et al. 2017). Several of these classes of therapeutic agents have been observed to partially restore pathophysiological changes in mitochondrial bioenergetics associated with AfSD in discrete CNS areas (Stefano and Kream 2015a, b). In contrast, there is a significant lack of empirical studies designed to evaluate the effects of commonly used antidepressant and neuroleptic agents on AfSD-associated gut dysbiosis (Macedo et al. 2017).

## Conclusion

Operationally, stereo-selective conformational matching between coupled physiological processes of bacterial microflora and intrinsic GI cells appear to support the conservation of a critically important set of chemical messengers required for existential regulation of homeostatic cellular processes within GI and CNS tissues (Stefano et al. 2017). Accordingly, it is not surprising to find that alterations in microbiome-based metabolic processes ultimately affect bidirectional communication between peripheral organs and CNS structures, thereby underlining the presence of potential common pathophysiological factors in psychiatric and GI disorders. Bacteria also have the ability to exchange their genomic information, providing a process for maintaining common signaling resulting in their selective survival (Lang and Beatty 2000). Alterations in essential ratios of gut enterotypes may directly affect activation states and cell surface markers of resident macrophages/microglia contained within CNS and ENS structures, thereby modulating bidirectional communication processes (Stefano et al. 1994) (Fig. 1). Finally, a more global view indicates a potential window of opportunity for development of novel therapeutic agents targeting microbiome, neurons, immune components, and the influence this has on brain function as a significant causative factor in severe behavioral disorders.

## Abbreviations

5HT: 5-hydroxytryptamine
AfSD: Affective spectrum disorders
CNS: Central nervous system
DA: Dopamine
DA-ergic: Dopaminergic
FOXG1: Forkhead box protein G1
GABA: Gamma-aminobutyric acid
GAD: Glutamic acid decarboxylase
GDAR: The glutamate-dependent acid resistance mechanism
GI: Gastrointestinal
IGF1: Insulin like growth factor 1
PD: Parkinson’s disease
PI3: Phosphoinositide 3
RTT: Rett syndrome
SCFA: Short chain fatty acids
SERT: Serotonin transporter
Tph: Tryptophan hydroxylase
